# Prevention of Image Quality Degradation in Wider Field Optical Coherence Tomography Angiography Images Via Image Averaging

**DOI:** 10.1167/tvst.10.13.16

**Published:** 2021-11-12

**Authors:** Kentaro Kawai, Akihito Uji, Takafumi Miyazawa, Tatsuya Yamada, Yuri Amano, Sonoka Miyagi, Ryangha Seo, Manabu Miyata, Shin Kadomoto, Akitaka Tsujikawa

**Affiliations:** 1 Department of Ophthalmology and Visual Sciences, Kyoto University Graduate School of Medicine, Kyoto, Japan

**Keywords:** optical coherence tomography angiography, image averaging, field of view, retinal imaging, image quality

## Abstract

**Purpose:**

To evaluate the mutual effect of widening the field of view and multiple en face image averaging on the quality of optical coherence tomography angiography (OCTA) images.

**Methods:**

This prospective, observational, cross-sectional case series included 20 eyes of 20 healthy volunteers with no history of ocular or systemic disease. OCTA imaging of a 3 × 3-mm, 6 × 6-mm, and 12 × 12-mm area centered on the fovea was performed nine times using the PLEX Elite 9000. We acquired averaged OCTA images generated from nine en face OCTA images. The corresponding areas in the three scan sizes were evaluated for the original single-scanned OCTA images and averaged OCTA images both qualitatively and quantitatively. Quantitative measurements included vessel density (VD), vessel length density (VLD), fractal dimension (FD), and contrast-to-noise ratio (CNR).

**Results:**

Significant differences in VD, VLD, FD, and CNR (*P* < 0.001) were observed due to the mutual effect of averaging and differences in scan size. Both qualitative and quantitative evaluations indicated that the quality of 6 × 6-mm averaged images was equal to or better than that of 3 × 3-mm single-scanned images. However, the quality of 12 × 12-mm averaged images did not reach that of 3 × 3-mm single-scanned images.

**Conclusions:**

To some extent, multiple en face OCTA image averaging can compensate for the deterioration in image quality caused by widening the field of view.

**Translational Relevance:**

Multiple en face OCTA image averaging can be a technique for acquiring wider field OCTA images with good quality.

## Introduction

Optical coherence tomography angiography (OCTA) is a recently developed technique that can isolate the microvascular circulation from OCT image data by detecting the motion contrast of blood flow without intravenous dye injections.^1^^–^^4^ Recent technologic advancements such as higher scan speed on swept-source (SS)-OCTA have enabled the capture of wider en face OCTA images than previously possible. Given the importance of evaluating morphological changes outside the macular area in patients with diseases such as diabetic retinopathy and retinal vein occlusion, there is a substantial need to develop OCTA techniques that increase the size of the scanning area. However, in general, a wider field of view yields wider spacing between A-scans. Therefore, the image quality or resolution of wider field OCTA is relatively lower than that of traditional macular OCTA.^5^ Acquiring wider field images with good image quality is necessary for the accurate diagnosis of peripheral lesions and accurate quantitative evaluation of peripheral lesions. For this reason, methods that enable more accurate and clearer visualization of vessels on wider field OCTA are desired.

Averaging of multiple en face OCTA images can make discontinuous vessel segments more continuous and reduce background noise, which can improve image quality for the retinal and choroidal microvasculature.^6^^–^^9^ Recent studies have shown that averaged OCTA images enable the observation of small microcirculation units known as arteriole–capillary–venule (ACV) units in the superficial retinal plexus and the meshwork structure of the choriocapillaris which are hardly detectable on single unaveraged OCTA images.^8^^,^^10^ However, to date, how OCTA averaging improves image quality and affects quantitative evaluation in wider field OCTA remains to be determined.

Therefore, in the present study, we assessed the impact of multiple en face image averaging on wider field OCTA images and aimed to investigate whether averaged multiple OCTA images can approximate the image quality of smaller field OCTA.

## Materials and Methods

This prospective, observational, cross-sectional case series was approved by the Institutional Review Board of the Kyoto University Graduate School of Medicine (Kyoto, Japan) and adhered to the tenets of the Declaration of Helsinki. Written informed consent was obtained from each volunteer prior to participation in the study.

### Participants

Twenty healthy volunteers with no history of ocular or systemic disease were recruited for the study. We analyzed data for one eye of each participant.

### OCTA Imaging

**Figure 1.**
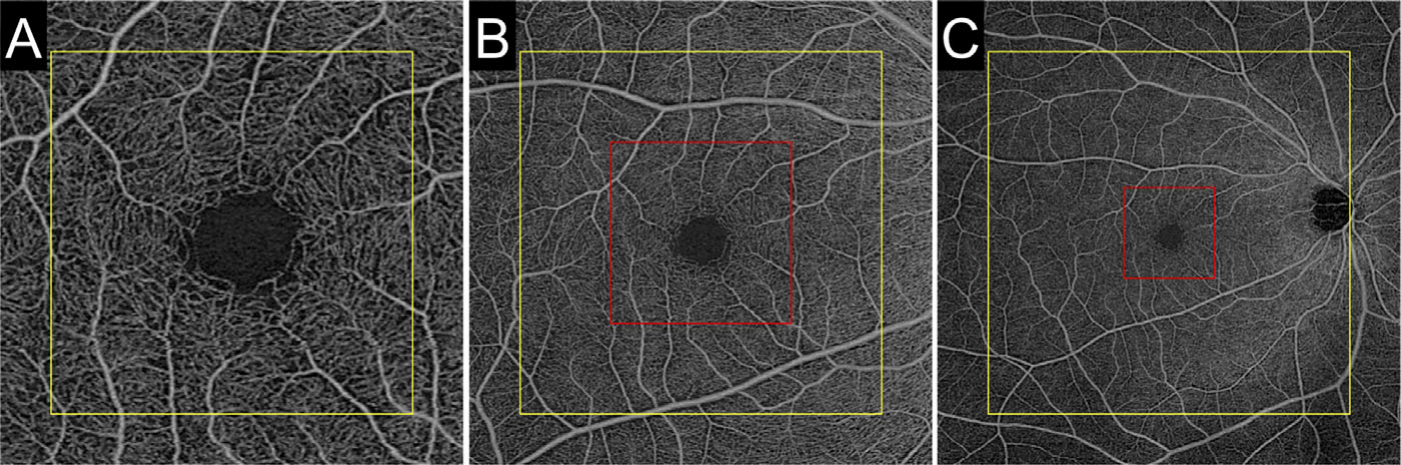
En face OCTA images of the superficial capillary plexus acquired using different scan sizes in the same participant: (**A**) 3 × 3-mm OCTA image; (**B**) 6 × 6-mm OCTA image; (**C**) 12 × 12-mm OCTA image. Shown are central square areas of 800 × 800 pixels (*yellow square* in A), 400 × 400 pixels (*red square* in **B**), and 200 × 200 pixels (*red square* in C) in a 3 × 3-mm OCTA image, a 6 × 6-mm OCTA image, and a 12 × 12-mm OCTA image, respectively. The central areas were subjected to qualitative and quantitative assessments.

The OCTA images were acquired using the PLEX Elite 9000 (Carl Zeiss Meditec, Dublin, CA). The PLEX Elite 9000 is a SS-OCTA instrument that uses a swept laser source with a central wavelength of 1040 to 1060 nm (980–1120 nm full bandwidth) and operates at 100,000 A-scans per second. For each participant, one eye was imaged using the 3 × 3-mm, 6 × 6-mm, and 12 × 12-mm scan protocols centered on the fovea over 3 days (the Supplementary Table shows information regarding each scan protocol). The examiners were instructed to scan repeatedly until nine OCTA scans of sufficient quality to meet prespecified acceptance criteria were obtained in each scan protocol. These acceptance criteria included clear and sharp focus, few to no artifacts (e.g., motion lines, blink artifacts), good centration, and signal strength of 7 or higher. En face images of the superficial capillary plexus (SCP; from the internal limiting membrane to the inner plexiform layer) and the deep capillary plexus (DCP; from the inner plexiform layer to the outer plexiform layer) were generated using the default setting of the manufacturer's software. The inner plexiform layer was defined as the layer at 70% of the inner thickness between the internal limiting membrane and the outer plexiform layer. All 3 × 3-mm, 6 × 6-mm, and 12 × 12-mm images of each layer were exported at a size of 1024 × 1024 pixels with the default brightness and contrast settings for further analysis (Fig. 1). Superficial vascular projections were removed using the projection removal algorithm of the instrument software before exporting the DCP images.

### Multiple En Face Image Averaging

A central square area of 800  ×  800 pixels was cropped for registration and averaging. Nine en face images from each layer were divided into nine sectors and subjected to both linear and nonlinear (elastic) image registration, following which the nine sectors were stitched back together. These steps were performed using ImageJ (National Institutes of Health, Bethesda, MD), as described in detail in our previous publication.^6^^,^^7^^,^^11^ Registration was first performed on the SCP, and then this same transformation information was applied to the DCP. After registration, the nine en face images from each layer were compounded into a single image by projecting the average intensity.

### Qualitative Assessment

**Figure 2.**
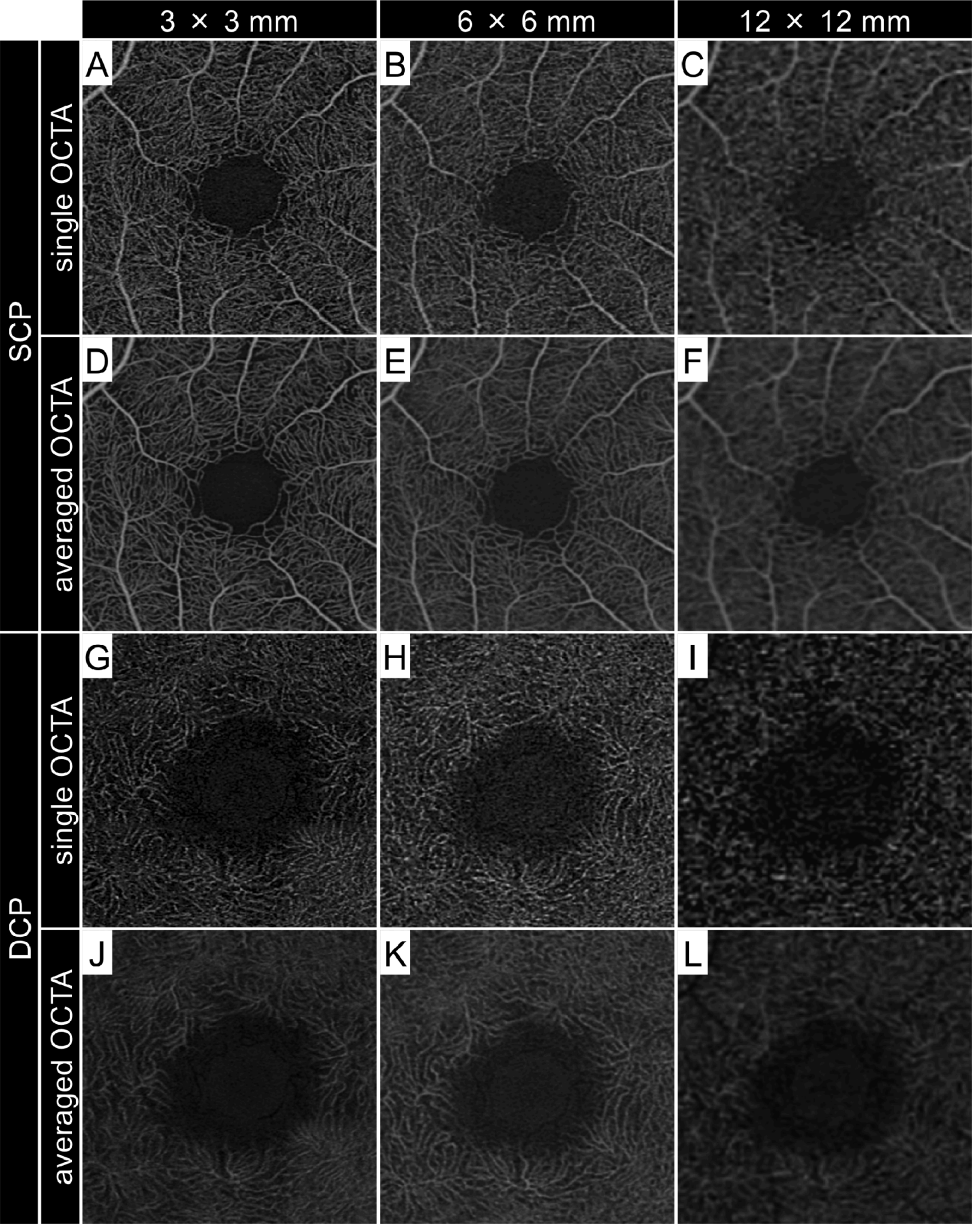
The central square area of OCTA images acquired using different scan sizes. (**A–F**) OCTA images of the SCP. (**G–L**) OCTA images of the DCP. (**A–C** and **G–I**) Single-scanned OCTA images. (**D–F** and **J–L**) Averaged OCTA images generated from nine en face OCTA images. (**A, D, G**, **J**) OCTA images with a scan size of 3 × 3 mm. (**B, E, H**, **K**) OCTA images scanned with a scan size of 6 × 6 mm. (**C, F, I**, **L**) OCTA images for a scan size of 12 × 12 mm.

Qualitative image assessments were performed by five graders (SM, TM, YA, RS, and TY). We used a paired comparison approach to assess OCTA image quality, as previously described.^6^^,^^7^ One case was randomly selected, and all combinations of averaged images and original unaveraged images of all scan protocols (3 × 3, 6 × 6, and 12 × 12-mm) were compared. A central square area of 400  ×  400 pixels in 6 × 6-mm images or 200  ×  200 pixels in 12 × 12-mm images was cropped to ensure comparison of the same area shown in 3 × 3-mm images (Fig. 2). Five graders performed independent comparisons of 30 pairs of OCTA images displayed horizontally in random order on a computer monitor. The graders were asked to compare image quality between pairs based on three parameters: (1) vessel quality (contrast and continuity), (2) nonvascular area quality (noise level), and (3) overall image quality. A comparative image quality score was assigned to each image pair as follows: 2 = substantially better quality for right image; 1 = slightly better quality for the right image; 0 = equal image quality; –1 = slightly better quality for the left image; and –2 = substantially better quality for the left image.

### Quantitative Measurements

**Figure 3.**
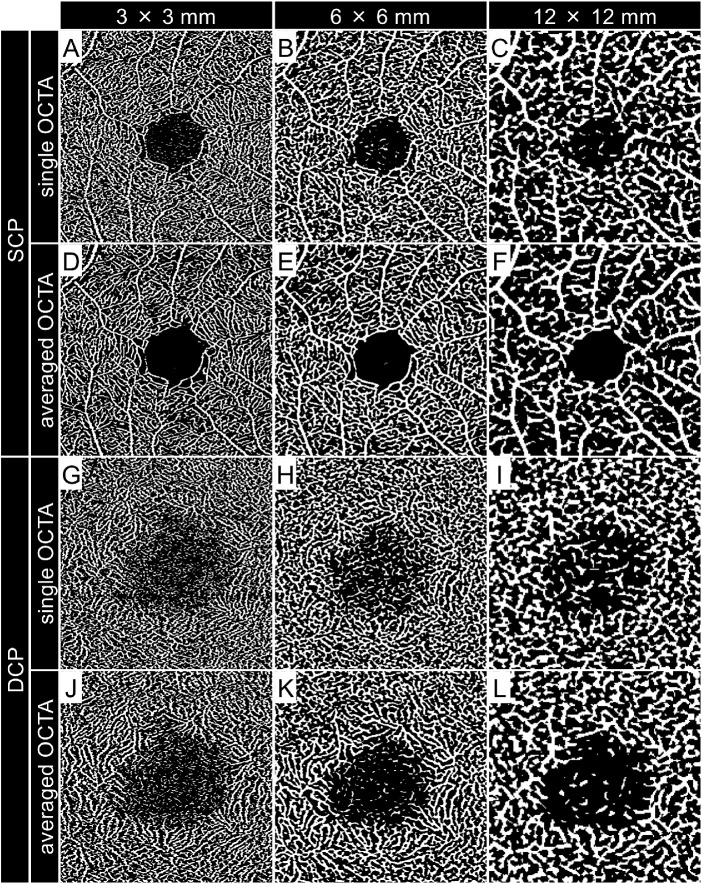
Central square area of binarized OCTA images acquired using different scan sizes. (**A–L**) Binarized OCTA images corresponding to **A** to **L** of Figure 2.

**Figure 4.**
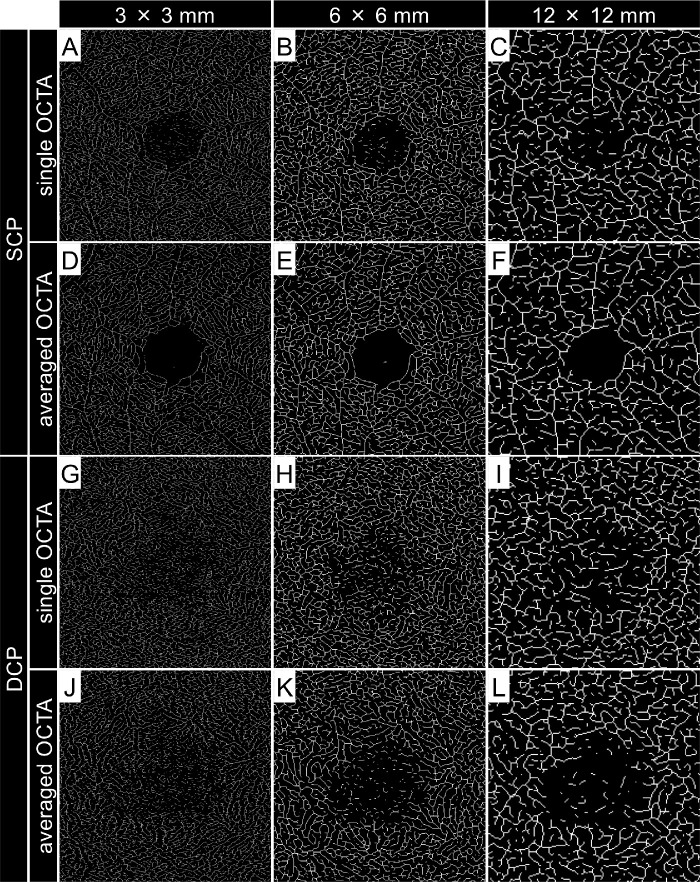
Central square area of skeletonized OCTA images acquired using different scan sizes. (**A–L**) Skeletonized OCTA images corresponding to **A** to **L** in Figures 2 and 3. In all images, *white segments* were eroded to a line with a single-pixel width.

Vessel density (VD), vessel length density (VLD), fractal dimension (FD), and contrast‐to‐noise ratio (CNR) were measured for both the SCP and DCP.^7^^,^^12^^–^^16^ The VD was calculated as the ratio of the area occupied by vessels divided by the total area of the binarized image. The VLD was calculated as the ratio of the area occupied by vessels divided by the total area of the skeletonized image. The averaged en face images were binarized using a modified version of the previously reported method.^1^^,^^2^^,^^7^ Briefly, after processing with a top-hat filter, the image was duplicated, and a different binarization method was performed. One image was processed first using a Hessian filter, followed by global thresholding using Huang's fuzzy thresholding method. The other (duplicate) image was binarized using median local thresholding. Then, the two different binarized images were combined to generate the final binarized image followed by skeletonization, reducing all of the continuous white segments to a line with a single-pixel width. The FD, which represents the complexity of the vasculature, was calculated on the skeletonized image using the box-counting method.^13^^,^^14^ The FD can range from 0 to 2, and images with a more complex vessel branching pattern will have a higher FD. After binarization and skeletonization, a central square area of 400  ×  400 pixels on 6 × 6-mm images and a central square area of 200  ×  200 pixels on 12 × 12-mm images were cropped to analyze the same areas observed on 3 × 3-mm images (Figs. 3, 4). These digital image processing steps were performed automatically by ImageJ and its plugin software.

For CNR calculation, a circular area within the foveal avascular zone (FAZ) was selected as the background region of interest (ROI), and the skeletonized vessels in the image were selected as the foreground ROI. For SCP images, parafoveal vessels of at least 100 pixels in length and bordering the FAZ were selected. For the DCP, because the vessels were fainter and less contiguous than those in the SCP, multiple vessels near the FAZ were selected to create foreground ROIs longer than 100 pixels in total length. The diameters of circular background ROIs within the FAZ were 25 pixels, 50 pixels, and 100 pixels in 3 × 3-mm scans, 6 × 6-mm scans, and 12 × 12-mm scans, respectively. The same background ROI was used for the SCP and DCP of the same scan size using ROI Manager, a built‐in function of ImageJ that records the exact location of the ROIs. Similarly, the positions of the foreground ROIs between single unaveraged OCTA images and averaged OCTA images were matched. The CNR was calculated as follows:

$$\begin{array}{c} \mathrm{CNR}=\left(f-b\right)/\sqrt{\delta_{f}^{2}+\delta_{b}^{2}}, \end{array}$$

where *f* and *b* are the mean gray values of the foreground and the background, respectively, and δ*_f_* and δ*_b_* are the standard deviations of the foreground and background gray values, respectively.

### Statistical Analysis

All values were expressed as the mean and standard deviation. Differences in VD, VLD, FD, and CNR were assessed using repeated-measures analysis of variance with Bonferroni correction. The scores generated by the graders were analyzed using Nakaya's variant of Scheffe's method of paired comparison. *P* < 0.05 was considered statistically significant. All analyses, except for Nakaya's, were performed using SPSS Statistics 24 (IBM, Armonk, NY). Nakaya's variant of Scheffe's method of paired comparison was performed using R 3.6.1 (R Foundation for Statistical Computing, Vienna, Austria).

## Results

The mean age of the included participants was 29.8 ± 10.1 years (range, 21–69). Ten participants were male, and ten were female. The Table shows the results of subjective image quality assessments. In all scan sizes, averaged OCTA images had significantly higher scores than single OCTA images of the same size, except for vessel quality and overall image quality of 12 × 12-mm SCP images. For all parameters, scores for the 3 × 3-mm averaged images were highest, followed by those for the 6 × 6-mm averaged images or 3 × 3-mm single images. The scores for 6 × 6-mm averaged images were higher than the scores for 3 × 3-mm single images for some parameters (overall image quality for SCP and DCP, vessel quality for DCP). There were no significant differences in the other parameters.

**Table.** Scores for Subjective Image Quality Assessment of Original Unaveraged OCTA Images and Averaged OCTA Images

	S3	A3	S6	A6	S12	A12	*Y* (0.05)^a^	*Y* (0.01)^b^
Superficial capillary plexus								
Vessel quality	0.53	1.47	–0.10	0.53	–1.27	–1.17	0.44	0.54
Nonvascular area quality	0.30	1.43	–0.37	0.53	–1.17	–0.73	0.31	0.38
Overall image quality	0.30	1.43	–0.10	0.63	–1.17	–1.10	0.33	0.39
Deep capillary plexus								
Vessel quality	0.03	1.30	–0.27	0.63	–1.20	–0.50	0.51	0.61
Nonvascular area quality	0.17	1.50	–0.70	0.53	–1.00	–0.50	0.46	0.56
Overall image quality	0.00	1.50	–0.43	0.70	−1.10	–0.67	0.38	0.46

S3, 3 × 3-mm single OCTA images; A3, 3 × 3-mm averaged OCTA images; S6, 6 × 6-mm single OCTA images; A6, 6 × 6-mm averaged OCTA images; S12, 12 × 12-mm single OCTA images; A12, 12 × 12-mm averaged OCTA images.

a Calculated yardstick for 5% significance level.

b Calculated yardstick for 1% significance level.

**Figure 5.**
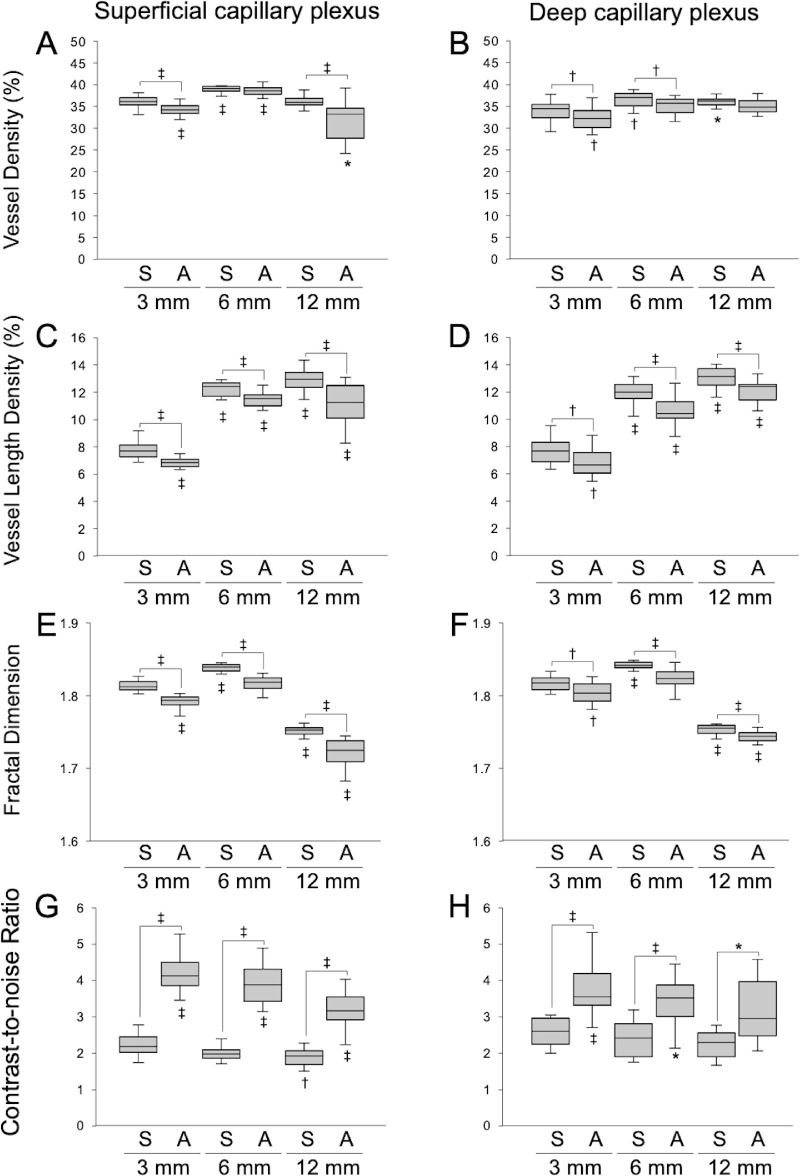
Differences in quantitative measurements between single-scanned OCTA images and averaged OCTA images acquired using different scan sizes. (**A, B**) Vessel density; (**C, D**) vessel length density; (**E, F**) fractal dimension; (**G, H**) contrast-to-noise ratio in the superficial capillary plexus (**A, C, E**, **G**) and the deep capillary plexus (**B, D, F**, **H**). There were significant differences in all parameters in both layers. Significant differences between groups of the same scan size in the same layer (above the box) and significant differences between each group and 3 × 3-mm single OCTA images (below the box) are indicated. S, single-scanned OCTA images; A, averaged OCTA images generated from nine OCTA scans. ^*^P < 0.05; ^†^*P* < 0.01; ^‡^*P* < 0.001.

The impact of scan size and averaging on microvascular parameters is shown in Figure 5. For both the SCP and DCP, significant differences in all parameters were observed among the groups. VLD was lowest for the 3 × 3-mm averaged images. For both the SCP and DCP, VLD and FD were lower in averaged images than in single images of the same size. Regarding VD, there was a significant difference between single images and averaged images in the 3 × 3-mm SCP scans, 12 × 12-mm SCP scans, 3 × 3-mm DCP scans, and 6 × 6-mm DCP scans. In the SCP, CNR was highest in 3 × 3-mm averaged images. (There was no significant difference between averaged 3 × 3-mm images and averaged 6 × 6-mm images.) CNR was higher in averaged images than single images of the same size. A similar tendency was observed in the DCP, although some of the pairs with significant differences in the SCP did not exhibit significant differences in the DCP.

## Discussion

In this study, we evaluated the mutual effect of widening the field of view and multiple en face image averaging on the quality of OCTA images. OCTA images of different sizes centered on the fovea were obtained from healthy eyes, and the effect of averaging was evaluated both subjectively and objectively. Our findings suggest that averaging enhances image quality even in wider field OCTA. Moreover, our analysis indicated that scan size and averaging exerted a significant impact on quantitative metrics.

Qualitative assessments indicated that, for all scan sizes, averaged OCTA images were given higher scores than single OCTA images of the same scan size. (However, there was no significant difference in vessel quality and overall image quality for 12 × 12-mm SCP images.) Several studies have demonstrated that averaging multiple 3 × 3-mm or 6 × 6-mm OCTA images improves image quality by increasing vessel continuity and reducing background noise.^6^^,^^7^^,^^11^^,^^17^ This finding is in accordance with the results of the present study, which further indicated that averaging multiple OCTA images is effective even in wider field OCTA. In our study, a larger field of view was associated with a lower image quality rating for all parameters. Meanwhile, the image quality of 6 × 6-mm averaged images was equal to or better than that of 3 × 3-mm single images. However, the image quality of the 12 × 12-mm averaged images was less than that of 3 × 3-mm single images for both the SCP and DCP and 6 × 6-mm single images for the SCP.

Previous studies have reported that differences in scan size affect the quantitative evaluation of vessels.^18^^,^^19^ Our results also indicate that averaging methods influence quantitative evaluation. For both the SCP and DCP, VLD was lower in averaged images than in single images of the same size. This result may reflect a decrease in background noise, which may have contributed to the decreased pixel number in the averaged images. The results of the qualitative assessment support this. Similarly, FD was lower in averaged images than in single images of the same size in both the SCP and DCP. Given that FD represents image complexity and background noise, and spiny vessel images contribute to an increase in FD,^7^ our results suggest that averaged images exhibit less background noise and smoother vessels than single images for all scan sizes.

In both the SCP and DCP, FD was significantly lower in 3 × 3-mm single images than in 6 × 6-mm single images and significantly higher in 3 × 3-mm single images than in 12 × 12-mm single images. This result suggests that 6 × 6-mm single images exhibit more background noise and rougher vessels than 3 × 3-mm single images, and that 12 × 12-mm single images depict less of the vasculature than 3 × 3-mm single images. On the other hand, there was no significant difference between the FD of 6 × 6-mm averaged images and 3 × 3-mm single images. This finding suggests that the quality of 6 × 6-mm images approached that of 3 × 3-mm images by averaging.

VLD was higher in 12 × 12-mm images than in 3 × 3-mm images, whereas the FD was lower in 12 × 12-mm images than in 3 × 3-mm images. When comparing VLD in the same area among images with different scan pattern sizes, attention should be paid to interpretation. In this study, central square areas of 800  ×  800 pixels, 400  ×  400 pixels, and 200  ×  200 pixels were cropped to ensure comparison of the same area in 3 × 3-mm images, 6 × 6-mm images, and 12 × 12-mm images, respectively. However, during skeletonization, white segments were eroded to a line with a single-pixel width for all scan sizes (Fig. 4). Thus, VLD was over-evaluated in skeletonized images with wide scan pattern sizes when compared with those with small scan pattern sizes. This may explain why the FD and VLD were not parallel.

In both the SCP and DCP, the CNR was higher in averaged images than in single images of the same size, in accordance with the results of the qualitative assessment. On the other hand, in the SCP, the CNR of 12 × 12-mm averaged images was higher than that of 3 × 3-mm single images, in contrast to the results of the qualitative assessment. The averaged images generated from multiple OCTA images exhibited small variations in gray values, which increased CNR. However, slight misalignment during averaging can blur the vessel boundaries, which did not affect CNR because skeletonized vessels were selected as the foreground ROI. This may explain in part the difference between the results of the qualitative and CNR assessments.

**Figure 6.**
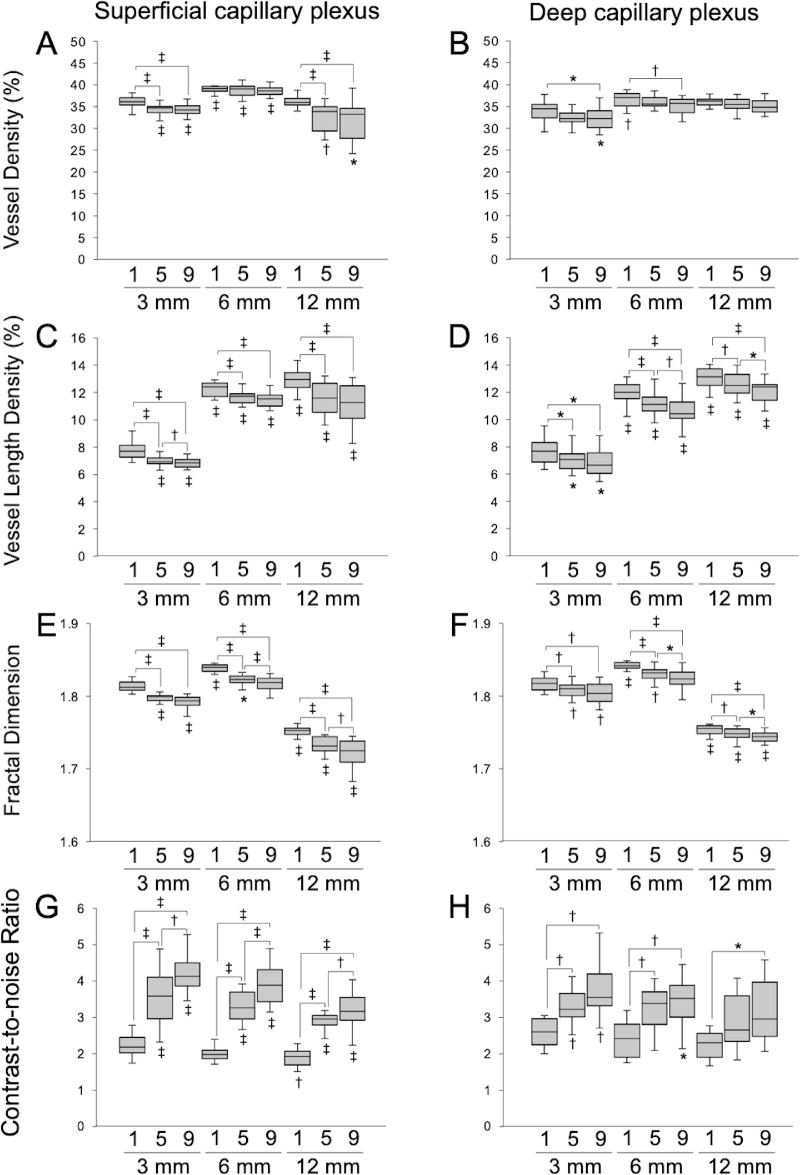
Differences in quantitative measurements due to the number of OCTA images used for averaging and the scan size. (**A, B**) Vessel density; (**C, D**) vessel length density; (**E, F**) fractal dimension; (**G, H**) contrast-to-noise ratio in the superficial capillary plexus (**A, C, E**, **G**) and the deep capillary plexus (**B, D, F**, **H**). Significant differences between groups of the same scan size in the same layer (above the box) and significant differences between each group and 3 × 3-mm single OCTA images (below the box) are indicated. 1, single-scan OCTA images; 5, averaged OCTA images generated from five OCTA scans; 9, averaged OCTA images generated from nine OCTA scans. ^*^P < 0.05; ^†^*P* < 0.01; ^‡^*P* < 0.001.

Despite the usefulness of OCTA image averaging, it is sometimes difficult to acquire a large number of OCTA images in a patient's eyes. Previous studies have shown that, although the image quality improved as the number of images used for averaging increased to nine, the differences were smaller after five images.^6^^,^^7^ We also investigated the quantitative measurements of averaged OCTA images generated from five scans (Fig. 6). Although the FD was slightly higher in 6 × 6-mm averaged images generated from five scans than in 3 × 3-mm single images, the FD was lower in averaged images generated from five scans than in single images, in all scan sizes, just like the averaged images generated from nine scans. If it is challenging to acquire nine images, it is considered that an improvement in image quality can be obtained even if the number of images used for averaging is reduced as necessary.

In recent years, most OCTA devices have incorporated montage techniques that stitch multiple OCTA images to acquire wider field images,^20^^,^^21^ and there are commercialized devices that have built-in software programs that average multiple OCTA images.^17^ Moreover, some commercialized OCTA devices include artificial intelligence (AI)-assisted denoising functions.^22^^,^^23^ Our findings demonstrate that the averaging technique is a valid option for improving image quality in wider field OCTA. However, further studies are required to determine whether averaging multiple wider field OCTA images, stitching multiple smaller images using a montage technique, or AI-assisted denoising of single wider field images is better for acquiring wider field OCTA images with good quality.

Our study has several limitations that should be considered when assessing our findings. First, our sample size was small, and the study included mostly young participants. Second, residual superficial projection artifacts may still have some effect on deeper layer imaging despite the use of projection removal software, as noted in a previous study.^24^ Finally, the accuracy of the image registration between scans may affect the quality of the averaged OCTA images.

In summary, our findings demonstrate that averaging multiple OCTA images can improve image quality for wider field OCTA. Although the image quality of 12 × 12-mm averaged images did not reach that of 3 × 3-mm single images, the image quality of 6 × 6-mm averaged images was comparable to that of 3 × 3-mm single images. Meanwhile, considering the significant differences in quantitative data between single unaveraged images and averaged images, quantitative image comparison between these two types of images is not recommended. Further study is warranted to confirm the usefulness of averaged wider field OCTA images as a replacement for single unaveraged OCTA images with a narrower field of view in patients with ocular diseases.

## Supplementary Material

Supplement 1

## References

[1] Jia Y, Tan O, Tokayer J, et al. Split-spectrum amplitude-decorrelation angiography with optical coherence tomography. *Opt Express*. 2012; 20(4): 4710–4725. 22418228 10.1364/OE.20.004710 PMC3381646

[2] Jia Y, Bailey ST, Hwang TS, et al. Quantitative optical coherence tomography angiography of vascular abnormalities in the living human eye. *Proc Natl Acad Sci USA*. 2015; 112(18): E2395–E2402. 25897021 10.1073/pnas.1500185112 PMC4426471

[3] Spaide RF, Fujimoto JG, Waheed NK, Sadda SR, Staurenghi G. Optical coherence tomography angiography. *Prog Retin Eye Res*. 2018; 64: 1–55. 29229445 10.1016/j.preteyeres.2017.11.003 PMC6404988

[4] Spaide RF, Klancnik JM Jr, Cooney MJ. Retinal vascular layers imaged by fluorescein angiography and optical coherence tomography angiography. *JAMA Ophthalmol*. 2015; 133(1): 45–50. 25317632 10.1001/jamaophthalmol.2014.3616

[5] Hirano T, Kitahara J, Toriyama Y, Kasamatsu H, Murata T, Sadda S. Quantifying vascular density and morphology using different swept-source optical coherence tomography angiographic scan patterns in diabetic retinopathy. *Br J Ophthalmol*. 2019; 103(2): 216–221. 29706601 10.1136/bjophthalmol-2018-311942

[6] Uji A, Balasubramanian S, Lei J, et al. Multiple enface image averaging for enhanced optical coherence tomography angiography imaging. *Acta Ophthalmol*. 2018; 96(7): e820–e827. 29855147 10.1111/aos.13740

[7] Uji A, Balasubramanian S, Lei J, et al. Impact of multiple en face image averaging on quantitative assessment from optical coherence tomography angiography images. *Ophthalmology*. 2017; 124(7): 944–952. 28318637 10.1016/j.ophtha.2017.02.006

[8] Uji A, Balasubramanian S, Lei J, Baghdasaryan E, Al-Sheikh M, Sadda SR. Choriocapillaris imaging using multiple en face optical coherence tomography angiography image averaging. *JAMA Ophthalmol*. 2017; 135(11): 1197–1204. 28983552 10.1001/jamaophthalmol.2017.3904 PMC5710392

[9] Mo S, Phillips E, Krawitz BD, Garg R, Salim S, Chui P. Visualization of radial peripapillary capillaries using optical coherence tomography angiography: the effect of image averaging. *PLoS One*. 2017; 12(1): e0169385. 28068370 10.1371/journal.pone.0169385 PMC5222511

[10] Muraoka Y, Uji A, Ishikura M, Iida Y, Ooto S, Tsujikawa A. Segmentation of the four-layered retinal vasculature using high-resolution optical coherence tomography angiography reveals the microcirculation unit. *Invest Ophthalmol Vis Sci*. 2018; 59(15): 5847–5853. 30535425 10.1167/iovs.18-25301

[11] Uji A, Balasubramanian S, Lei J, Baghdasaryan E, Al-Sheikh M, Sadda SVR. Choriocapillaris imaging using multiple en face optical coherence tomography angiography image averaging. *JAMA Ophthalmol*. 2017; 135(11): 1197–1204. 28983552 10.1001/jamaophthalmol.2017.3904 PMC5710392

[12] Kim AY, Chu Z, Shahidzadeh A, Wang RK, Puliafito CA, Kashani AH. Quantifying microvascular density and morphology in diabetic retinopathy using spectral-domain optical coherence tomography angiography. *Invest Ophthalmol Vis Sci*. 2016; 57(9): OCT362–OCT370. 27409494 10.1167/iovs.15-18904 PMC4968771

[13] Reif R, Qin J, An L, Zhi Z, Dziennis S, Wang R. Quantifying optical microangiography images obtained from a spectral domain optical coherence tomography system. *Int J Biomed Imaging*. 2012; 2012: 509783. 22792084 10.1155/2012/509783 PMC3389716

[14] Zahid S, Dolz-Marco R, Freund KB, et al. Fractal dimensional analysis of optical coherence tomography angiography in eyes with diabetic retinopathy. *Invest Ophthalmol Vis Sci*. 2016; 57(11): 4940–4947. 27654421 10.1167/iovs.16-19656

[15] Stetson PF, Sommer FG, Macovski A. Lesion contrast enhancement in medical ultrasound imaging. *IEEE Trans Med Imaging*. 1997; 16(4): 416–425. 9262999 10.1109/42.611351

[16] Uji A, Ooto S, Hangai M, Arichika S, Yoshimura N. Image quality improvement in adaptive optics scanning laser ophthalmoscopy assisted capillary visualization using B-spline-based elastic image registration. *PLoS One*. 2013; 8(11): e80106. 24265796 10.1371/journal.pone.0080106 PMC3827159

[17] Uji A, Sadda SR, Muraoka Y, et al. Effect of image averaging on optical coherence tomography angiography data in eyes with branch retinal vein occlusion. *Graefes Arch Clin Exp Ophthalmol*. 2020; 258(8): 239–248. 10.1007/s00417-020-04713-9 32361802

[18] Rabiolo A, Gelormini F, Marchese A, et al. Macular perfusion parameters in different angiocube sizes: does the size matter in quantitative optical coherence tomography angiography? *Invest Ophthalmol Vis Sci*. 2018; 59(1): 231–237. 29340651 10.1167/iovs.17-22359

[19] Lei J, Durbin MK, Shi Y, et al. Repeatability and reproducibility of superficial macular retinal vessel density measurements using optical coherence tomography angiography en face images. *JAMA Ophthalmol*. 2017; 135(10): 1092–1098. 28910435 10.1001/jamaophthalmol.2017.3431 PMC5710485

[20] Sawada O, Ichiyama Y, Obata S, et al. Comparison between wide-angle OCT angiography and ultra-wide field fluorescein angiography for detecting non-perfusion areas and retinal neovascularization in eyes with diabetic retinopathy. *Graefes Arch Clin Exp Ophthalmol*. 2018; 256(7): 1275–1280. 29713816 10.1007/s00417-018-3992-y

[21] Kadomoto S, Uji A, Muraoka Y, Akagi T, Miyata M, Tsujikawa A. A novel strategy for quantification of panoramic en face optical coherence tomography angiography scan field. *Graefes Arch Clin Exp Ophthalmol*. 2019; 257(6): 1199–1206. 30972485 10.1007/s00417-019-04310-5

[22] Kadomoto S, Uji A, Muraoka Y, Akagi T, Tsujikawa A. Enhanced visualization of retinal microvasculature in optical coherence tomography angiography imaging via deep learning. *J Clin Med*. 2020; 9(5): 1322. 10.3390/jcm9051322 PMC7290309 32370282

[23] Sawai Y, Miyata M, Uji A, et al. Usefulness of denoising process to depict myopic choroidal neovascularisation using a single optical coherence tomography angiography image. *Sci Rep*. 2020; 10(1): 6172. 32277172 10.1038/s41598-020-62607-6 PMC7148361

[24] Braun PX, Mehta N, Gendelman I, et al. Global analysis of macular choriocapillaris perfusion in dry age-related macular degeneration using swept-source optical coherence tomography angiography. *Invest Ophthalmol Vis Sci*. 2019; 60(15): 4985–4990. 31791062 10.1167/iovs.19-27861 PMC6890395

