# Supplementary material for: Prevention of Image Quality Degradation in Wider Field Optical Coherence Tomography Angiography Images Via Image Averaging
Source: Transl Vis Sci Technol. 2021 Nov 12;10(13):16. doi: 10.1167/tvst.10.13.16 (PMC8590158; doi:10.1167/tvst.10.13.16)
Supplement: Supplement 1 [file tvst-10-13-16_s001.pdf]

Supplementary Table. Information of scan protocols

Field of view	A-scans (n)	B-scans (n)	Resolution (μm)
3 × 3 mm	300	300	10
6 × 6 mm	500	500	12
12 × 12 mm	500	500	24
